# The association of different types of stress, and stress accumulation with low back pain in call-center workers - a cross-sectional observational study

**DOI:** 10.1186/s12891-024-08087-5

**Published:** 2024-11-28

**Authors:** Michael Brenner-Fliesser, Sanne Houtenbos, Marie Ewerton, Carolin Bontrup, Rosa Visscher, William R. Taylor, Roland Zemp, Pia-Maria Wippert

**Affiliations:** 1 LIFE – Institute for Climate, Energy and Society, Joanneum Research, Graz, 8020 Austria; 2 Medical Sociology and Psychobiology, Department of Health and Physical Activity, University of Potsdam, 14469 Potsdam, Germany; 3 Institute of Public Health, Zurich University of Applied Sciences (ZHAW), Winterthur, 8400 Switzerland; 4 Careum Hochschule Gesundheit, Kalaidos Fachhochschule, Zürich, 8006 Switzerland; 5 Laboratory for Movement Biomechanics, Department of Health Sciences and Technology, Institute for Biomechanics, ETH Zürich, 8093 Zürich, Switzerland; 6 Faculty of Health Sciences Brandenburg, Joint Faculty, University of Potsdam, the Brandenburg Medical School Theodor Fontane and the Brandenburg University of Technology Cottbus-Senftenberg, 14469 Potsdam, Germany

**Keywords:** Low back pain, Stress, Call-center workers, Sitting work, Stress types, Hair-cortisol

## Abstract

**Background:**

Low back pain (LBP) is a common health complaint and a prominent factor in the development of LBP among the working population is stress. Mostly, stress is addressed as a general problem, which is why LBP prevention programs are often imprecise. Accordingly, a closer look at the association between specific stress types and the development of LBP is necessary. Therefore, this paper aims (1) to identify the stress types most closely associated with LBP; (2) to examine the relationship between stress accumulation and LBP.

**Methods:**

*n* = 100 call-center workers were approached for participation. Stress levels and LBP were assessed with questionnaires (TICS, ERI, CPG, BPI) and hair cortisol levels were measured (ELISA-KIT, 3-months period). Mann-Whitney U tests were used to identify stress types most closely associated with LBP. Further, ANCOVA analysis was conducted to determine the association of the number of experienced stress types with LBP intensity and impairment.

**Results:**

Finally, data from *n* = 68 participants (mean age: 43.2 (± 12.8) years; 62% female) were used for presented analysis. Participants, who were affected by work-related stress showed higher pain severity (excessive demands at work: 23.6 ± 21.8 vs. 42.4 ± 25.0 (*p* = 0.005)) and more impairment (excessive demands at work: 13.7 ± 17.6 vs. 28.7 ± 22.3 (*p* = 0.003); work overload: 15.4 ± 20.4 vs. 26.3 ± 17.4 (*p* = 0.009)) than their less affected colleagues. Other stress types (e.g. Effort, Reward) showed no significant association with LBP. Furthermore, participants who experienced two or more of the most associated stress types simultaneously suffered from stronger pain and more impairment (*p* < 0.01).

**Conclusions:**

The results suggest that it is essential to divide and evaluate stress in specific domains. Furthermore, the accumulation of different stress types and the resulting physiological load should be taken into account when designing prevention and intervention programs. Results may be of high relevance for the development of LBP prevention programs for people within a predominantly sitting working context.

## Background

According to the Global Burden of Disease Study of 2019, low back pain (LBP) is one of the top causes of disability, placing a great cost burden on the healthcare society and patients [1, 2]. The majority of people will experience an episode of LBP at least once in their life [3]. Low back pain can consist of a range of pain types, including neuropathic, nociceptive and nociplastic pain [3]. The development of LBP is dependent on several factors, categorized in biological, psychological and social categories, as part of the biopsychosocial model [3, 4]. Examples of factors included in the biopsychosocial model are distress, pain-related cognitions, unfavorable cognitive beliefs, unsupportive workplaces, a sedentary lifestyle, and obesity [4–6]. All these factors have an influence on LBP; further, an accumulation of these factors may lead to a higher vulnerability for developing LBP [7, 8]. Unfortunately, some of these factors are almost unavoidable in specific life contexts [9]. For example, 39% of the working population in the EU spends a high proportion of their working hours seated [10]. This could pose an issue, since long sitting periods (over seven hours per day) have shown to be associated with LBP [11–13], even though the exact role of sitting behavior (e.g. time sitting, sitting position, etc.) in developing LBP still has to be clarified.

Commonly, an emphasis is put on the biological/biomedical aspect of the biopsychosocial model, and social domains are overlooked [14, 15]. However, a highly prominent factor in the development of LBP in the working context is stress [8]. Previous research has suggested work related stress, social isolation and job satisfaction as important factors to take into account when applying the biopsychosocial model approach [14]. Multiple studies have detected a connection between psychological distress at work and musculoskeletal pain (e.g. LBP) among several working populations, including office workers [16–21]. Since experiencing multiple psychosocial risk factors increases the chance of developing LBP, the risk of developing LBP may be even more pronounced in office workers, due to prolonged sitting and possible stress.

Various work-related stressors can have an influence on LBP. Previous studies have shown that especially high workload, low job satisfaction and decision latitude are correlated with LBP among office workers [22–24]. Furthermore, an association between high job demands, low control and musculoskeletal pain in female VDU (visual display unit) operators was reported [25]. While it is plausible that employees with a high stress load have a higher risk of developing LBP, prevention programs focusing on stress reduction and management show mixed results [26] or seem to be inefficient [27]. This may be due to the fact that most prevention programs aim to reduce stress in general [26]. Since not all types of stress are associated with LBP equally [28, 29], these general prevention programs may not lead to the desired results. It may be more beneficial to distinguish between different subtypes of stress (e.g. work overload, social overload, lack of social recognition) and to target the factors most strongly related to LBP. A previous study, including a population suffering from back pain, found that tendency to worry, vital exhaustion, social isolation, and the occurrence of life events influenced characteristic pain intensity, whereas work discontent, social isolation, tendency to worry and life events foreshowed pain-related disability [29]. Moreover, it may be important to have a closer look at the accumulation of different stress types within one person [8, 30, 31]. The accumulation of multiple stress types leads to upregulated activity of allosteric systems, characterized by the allostatic load concept, making an individual more vulnerable to developing chronic pain [8, 31, 32].

Hence, this contribution will expand the current state of research in two ways: Firstly, stress will be classified in various types and be measured subjectively as well as objectively to evaluate its association with LBP in a sample of call-center office workers. According to previous work analysing 26 occupations, call-center work is among the six most stressful occupations, possibly due to a lack of control over working procedures [33]. Secondly, it will be evaluated if employees experiencing different types of stress at the same time report stronger and more disabling LBP than their less affected colleagues. In detail, two hypotheses should be analyzed:

H1: The association of various individual stress types with LBP intensity and disability differs among call-center workers, whereby work-related stress is an important factor.

H2: Call-center workers aggregating multiple stress types report stronger and more disabling LBP than their less affected colleagues.

## Materials and methods

### Design

This cross-sectional study was conducted at two worksites of a call-center company in North-East Germany. The working task itself is highly standardized and includes answering customer inquiries and complaints for a mail-order firm (inbound marketing) via phone and e-mail. During the two weeks of investigation, a team of scientist from the University of Potsdam and ETH Zürich were stationed locally to perform measurements, conduct questionnaires and answer participant questions. For each participant, the answering of questionnaires (either in their breaks or at the end of their working day) regarding stress and the taking of hair samples were completed in one day and conducted by trained staff of either the University of Potsdam and/or the ETH Zürich. Furthermore, motion sensors were used to record measurements of the sitting position and movement behavior [34]. After completion of all questionnaires and measurements, participants received 15 Euros as compensation and were provided with their individual results from their questionnaires and hair sample analysis. The study was conceptualized and performed in accordance with the principles of the Declaration of Helsinki and was approved by the ethics committee of the University of Potsdam, Germany (no. 42/2014), with the confirmation of the ethics committee of the ETH Zürich, Switzerland.

### Participants

Call-center employees from two call-center locations (Leipzig *n* = 70; Dresden *n* = 80 (Germany)) with standardized working tasks and process, were approached for participation. To be eligible for participation, participants had to be between 18 and 65 years old and fluent in German. Exclusion criteria were being pregnant, consumption of glucocorticoids (possible distortion of hair cortisol measurement) or currently receiving medical treatment for other reasons than back pain (e.g. metabolic diseases). All participants signed the informed consent form.

A sample size calculation based on pilot study data was performed using the statistical software G*Power 3.1. Based on mean values and standard deviations included in the pilot study (*n* = 20 [35], , with an *effect size of 1.02* and a *power of 0.96*, a required sample size of 58 was revealed (two-tailed independent samples Mann-Whitney-U test).

### Instruments

*Sociodemographic* data, as well as data regarding *health*,* medication and physical activity* were collected through questionnaires.

#### Psychometric measures

*Chronic Stress* was assessed by the Trier Inventory for Chronic Stress (TICS) [36], consisting of 57 items rated on a 5-point Likert scale (from 0 = „never“ to 4 = „very often“). Each item indicates how often someone experienced a certain stressful situation within the past three months. The items are summed up to nine scales (*work overload*,* social overload*,* pressure to perform*,* work discontent*,* excessive demands from work*,* lack of social recognition*,* social tensions*,* social isolation*,* chronic worrying)* giving information about the stress load in various areas.

*Stress at work* was measured using the Effort Reward Imbalance questionnaire (ERI) [37]. This questionnaire consists of 16 items measuring e*ffort*,* reward (subscales: esteem*,* job promotion*,* job security)* and *overcommitment* on a 4-point Likert scale (from 0 = “not true at all” to 3 = “completely true”). An additional scale, the effort-reward ratio, associates effort with reward and calculates if the effort is higher than the reward. Two scales (job promotion and job security) were deleted from further analysis since their Cronbach’s *α* levels were not acceptable (Cronbach’s *α* levels < 0.65).

*Chronic low back pain* over the past three months was assessed using the Chronic Pain Grade Questionnaire (CPG) and acute low back pain in the last 24 h was assessed using the Brief Pain Inventory (BPI) [38, 39]. The CPG consists of seven questions divided over two subscales: pain intensity (CPI) and disability (DISS) over the past three months. Each item ranged from 0 (no pain or impairment) to 10 (worst possible pain or I wasn’t able to do anything [34].

The BPI estimated the acute pain in the past 24 h and was also divided into two subscales: pain severity and pain-related interference of daily functions. Each item could be answered on a range from 0 to 10, with 0 (no pain/no interference) to 10 (pain as bad as you can imagine/interferes completely). The BPI also includes a body chart, on which the participants were required to indicate the location of their pain.

All questionnaires used to assess psychometric measures and back pain mentioned above were validated for the German language. The data gathered through questionnaires and measurements were code-protected and finally anonymized.

#### Biomarkers

Hair cortisol was used as a physiological measure of stress. Therefore, two hair strands of a length of at least three centimeters (diameter approximately 2 mm) were cut from the back of the head below the covering hair and analyzed by ELISA (IBL, RE62019). Hair cortisol samples were only collected if the hair was at least 3 centimeters long, indicating the stress level of the last three months [40]. Hair cortisol levels were measured in pg/mg.

### Data preprocessing and statistical analysis

Based on their individual scores, participants were divided into tertiles (low, medium, high) for each psychometric stress scale. The same was done for the hair cortisol measurements on basis of the individual cortisol level detected. If their scores/cortisol level fell within the highest tertile of the distribution the participants were labeled as “highly burdened by stress”. For the reward scales, participants were labeled as highly burdened by stress, if they fell within the lowest tertile of the distribution.

Afterwards, Mann-Whitney U tests were conducted for each stress type to analyze if highly burdened participants suffer from significantly stronger and more disabling LBP than less affected participants (hypothesis 1). In this case, an effect size of *r* ≥ 0.3 (medium to strong effect [41]) is considered as relevant. To compensate for multiple testing, the significance levels were reduced to *p* = 0.006 for the nine TICS scales (Bonferroni correction: $\:p=\frac{\alpha\:}{n}$) and to *p* = 0.01 for the five ERI scales.

To investigate whether participants affected by various stress types simultaneously suffer from stronger and more disabling LBP than participants whoare less affected, the three stress types with the biggest differences between the low- and high-burdened groups were added up. Analysis of Covariance (ANCOVA) with planned contrasts was used to compare pain intensity and disability between participants who belonged to no, one, two or three of the highly burdened groups, while controlling for age and sex. Statistical significance for the ANCOVA was set at *p* < 0.05. All available data were used in the analysis, and participants with missing values regarding the CPG questionnaire were included to enhance the robustness of our findings.

## Results

**Fig. 1:**
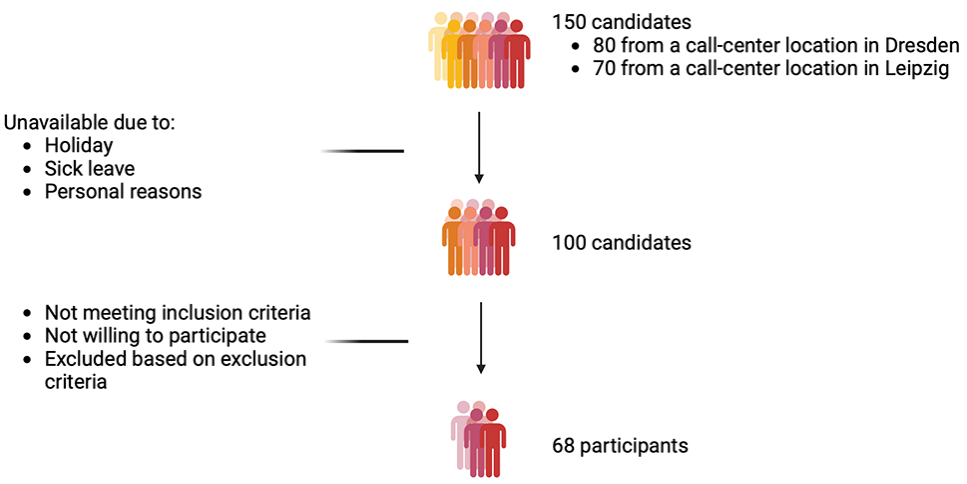
Participant recruitment flow chart. Created in BioRender. Houtenbos, S. (2024)

Out of 150 candidates, 50 were unavailable due to sick leave, holidays, etc., leading to a total of 100 candidates who were approached for participation in this study. Finally, a sub-sample of *n* = 68 participants were included for the presented study objectives (Fig. 1).

The average (mean ± SD) age of included participants was 43.2 ± 12.8 years and 62% (*n* = 42) were female (Table 1). Participants were, on average, highly educated (median: upper secondary education), mainly inactive in physical sports (66%) with a mean household income of 1799 (*SD* = 1125) Euros per month. The average chronic pain intensity (28.7 ± 24.0) as well as chronic pain disability (18.1 ± 20.1) was low [38]. Significant differences due to age, sex, education, and income were present for work discontent (education: secondary school with vocational training vs. high school diploma (A-levels) without/with vocational training; *p* < 0.05), social conflicts (age: 46.27 ± 12.42 vs. 36.67 ± 11.40; *p* < 0.05), social isolation (education: secondary school with vocational training vs. high school diploma (A-levels) without/with vocational training, *p* < 0.05; income: €2021.28 ± 1193.73 vs. €1344.74 ± 824.00; *p* < 0.05) and reward esteem (66.07% vs. 30.00% female; *p* < 0.05). Highly burdened participants were, on average, higher educated and younger than less affected participants.

**Table 1:** Participant characteristics for *n* = 68 included participants

Variable	*N*	Mean ± SD
Sex (M/F) (%F)	68	26/42 (62%)
Age (y)	66	43.2 ± 12.8
BMI (kg/m^2^)	64	26.7 ± 6.2
Education (median)	62	Upper secondary education
Income (per month)	58	€1799 ± 1125
Regularly physical active (yes/no; %)	67	23/44 (33.8%)
Average CPI	66	28.7 ± 24.00
Average DISS	68	18.1 ± 20.1

### Hypothesis 1

Results predominantly show differences in LBP when assessing workers affected by specific work-related stress types compared to unaffected participants. The factor ‘excessive demands at work’ reported significantly stronger characteristic pain intensity (CPI) than less burdened participants (*r*=-0.34; *p* = 0.005). The other two stress types that indicate (non-significant) differences between highly burdened and less burdened groups, out of the tested markers with CPI are hair cortisol (*r*=-0.24; *p* = 0.14) and social tensions (*r*=-0.23; *p* = 0.07, see Table 2).

**Table 2:** Differences in characteristic low back pain intensity (CPI) between participants highly burdened and less burdened by different stress types

Stress type	Highly burdened	*N*	LBP intensity (0-100)		*p*	U	z	*r*
			M	SD				
***Trier Inventory for Chronic Stress***								
Work overload	No	51	27.1	25.2	0.19	297.5	-1.31	− 0.16
	Yes	15	34.4	19.4				
Social overload	No	45	28.1	24.5	0.63	438.0	-0.48	− 0.06
	Yes	21	30.0	23.6				
Pressure to perform	No	51	28.0	25.1	0.58	346.5	-0.56	− 0.07
	Yes	15	31.1	20.7				
Work discontent	No	48	27.4	24.4	0.44	378.5	-0.78	− 0.10
	Yes	18	32.4	23.3				
Excessive demands at work	**No**	**48**	**23.6**	**21.8**	0.005*	**239.5**	**-2.80**	**− 0.34**
	**Yes**	**18**	**42.4**	**25.0**				
Lack of social recognition	No	54	27.5	24.0	0.34	267.5	-0.95	− 0.12
	Yes	12	34.2	24.4				
Social tensions	No	46	24.9	23.1	0.07	329.0	-1.85	− 0.23
	Yes	20	37.5	24.5				
Social isolation	No	47	28.4	25.4	0.81	429.5	-0.24	− 0.03
	Yes	19	29.5	20.9				
Chronic worrying	No	50	27.5	22.9	0.55	360.5	-0.60	− 0.07
	Yes	16	32.7	27.8				
***Effort Reward Imbalance***								
Effort	No	44	26.7	23.8	0.56	358.5	-0.59	− 0.07
	Yes	18	30.9	25.7				
Reward	No	45	31.3	25.2	0.44	272.0	-0.77	− 0.10
	Yes	14	25.2	22.9				
Subscale esteem	No	56	29.1	22.1	0.55	194.5	-0.60	− 0.08
	Yes	8	28.9	37.4				
Overcommitment	No	49	26.4	24.7	0.22	290.0	-1.24	− 0.16
	Yes	15	33.6	21.5				
Effort-reward imbalance	No	39	26.6	23.8	0.26	285.5	-1.14	− 0.04
	Yes	18	34.4	25.6				
***Objectively measured stress***								
Hair cortisol level	No	28	25.8	23.6	0.14	128.0	-1.53	-0.24
	Yes	13	39.0	26.6				

* Bonferroni corrected α = 0.006 (TICS); α = 0.01 (ERI), Range of characteristic pain intensity (CPI): 0-100

Participants who experience excessive demands at work (*r*=-0.36; *p* = 0.003) report, on average, higher pain-related disability (DISS) than less burdened participants (Table 3). This is also seen for work overload (*r*=-0.32; *p* = 0.009), social tensions (*r*=-0.27; *p* = 0.03) and overcommitment (*r*=-0.24; *p* = 0.05), although lacking the Bonferroni-corrected significance level.

**Table 3:** Differences in low back pain-related disability (DISS) between participants highly burdened and less burdened by different stress types

Stress type	Highly burdened	*N*	LBP disability (0-100)		*p*	U	z	*r*
			M	SD				
***Trier Inventory for Chronic Stress***								
Work overload	No	51	15.4	20.4	0.009	254.5	-2.60	− 0.32
	Yes	17	26.3	17.4				
Social overload	No	46	16.7	20.4	0.31	431.0	-1.0	− 0.12
	Yes	22	20.9	19.7				
Pressure to perform	No	51	16.8	19.4	0.26	356.5	-1.12	− 0.14
	Yes	17	22.0	22.4				
Work discontent	No	48	15.4	17.3	0.17	380.0	-1.38	− 0.16
	Yes	20	24.5	25.1				
Excessive demands at work	**No**	**48**	**13.7**	**17.6**	0.003*	**266.5**	**-2.95**	**− 0.36**
	**Yes**	**20**	**28.7**	**22.3**				
Lack of social recognition	No	54	16.4	18.6	0.28	309.0	-1.08	− 0.13
	Yes	14	24.5	25.0				
Social tensions	No	46	14.5	17.8	0.03	339.5	-2.24	− 0.27
	Yes	22	25.6	23.1				
Social isolation	No	47	17.0	19.7	0.38	428.5	-0.89	− 0.11
	Yes	21	20.5	21.5				
Chronic worrying	No	50	16.2	19.1	0.18	356.5	-1.34	− 0.16
	Yes	18	23.3	22.6				
***Effort Reward Imbalance***								
Effort	No	45	17.9	20.3	0.67	399.0	-0.43	− 0.05
	Yes	19	19.1	20.3				
Reward	No	45	19.0	19.9	0.78	343.5	-0.28	− 0.04
	Yes	16	18.1	21.6				
Subscale esteem	No	56	18.0	18.9	0.99	279.0	-0.02	− 0.00
	Yes	10	22.0	27.4				
Overcommitment	No	50	16.2	20.9	0.05	273.0	-1.96	− 0.24
	Yes	16	23.8	17.3				
Effort-reward imbalance	No	39	16.8	20.1	0.25	320.0	-1.15	− 0.15
	Yes	20	21.7	20.2				
***Objectively measured stress***								
Hair cortisol level	No	28	16.1	19.4	0.70	142.0	-1.15	-0.18
	Yes	13	26.4	24.9				

* Bonferroni corrected α = 0.006 (TICS); α = 0.01 (ERI), Range of pain-related disability (DISS): 0-100

### Hypothesis 2

**Fig. 2:**
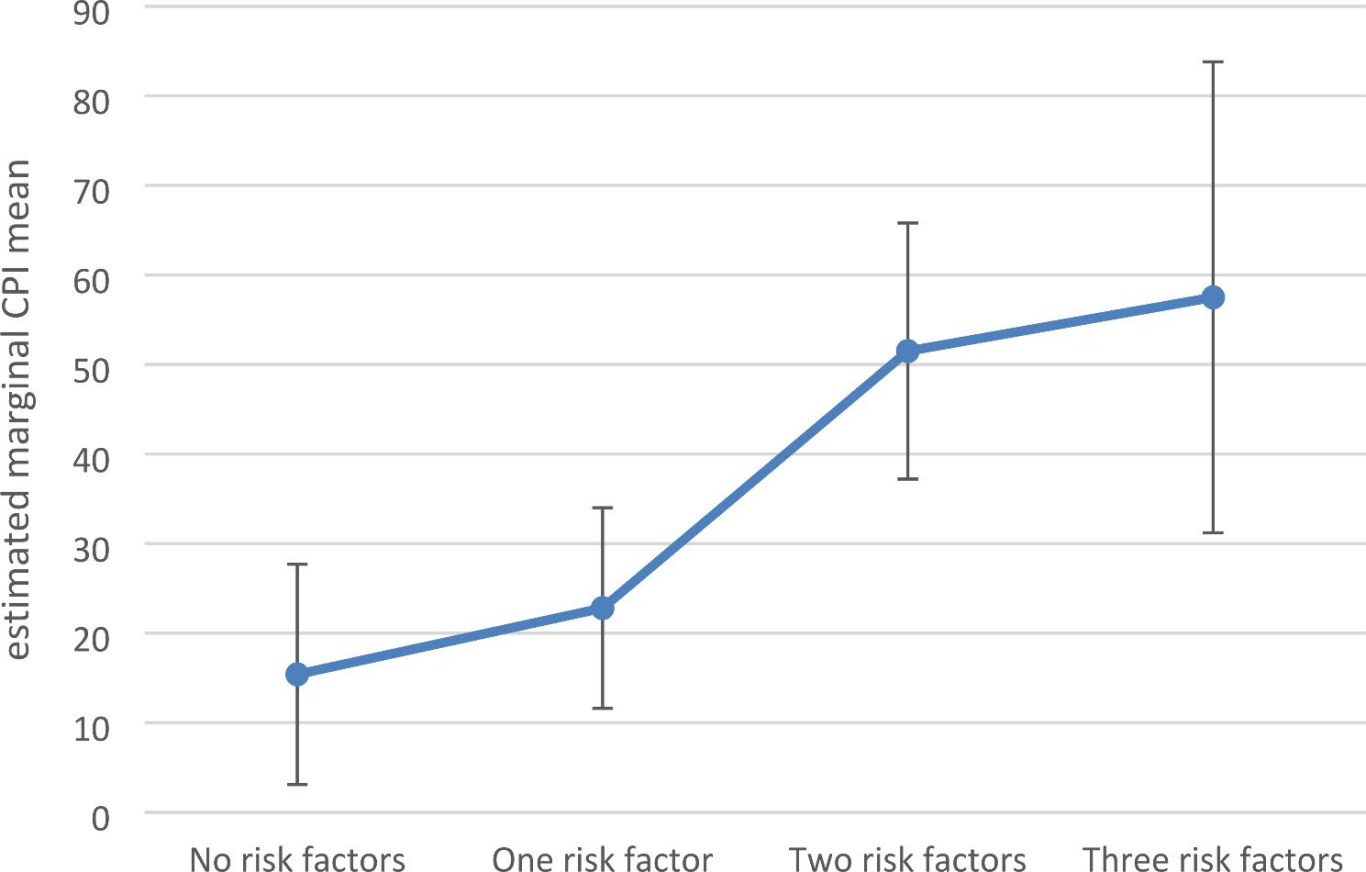
Estimated marginal means of characteristic pain intensity (CPI) as a function of the number of stress types (excessive demands at work, hair cortisol level and social tensions). Controlled for age and sex. *N* = 66. Bars indicate 95% CI

Taking into account the accumulation of the most associated stress types regarding CPI (hypothesis 1; excessive demands at work, hair cortisol level and social tensions), ANCOVA reveals that the number of stress types (0, 1, 2 or 3) is significantly associated with the extent of CPI (F 3, 35 = 6.8, *p* = 0.001, ω_p_²=0.309), whereas the covariates yield no significant difference. Planned contrasts reveal that, in comparison with no risk, being burdened in two (*p* < 0.001; 95% CI [16.6; 55.5]) or three (*p* = 0.006; 95% CI [12.9; 71.2]) of the most associated stress areas leads to a significant increase in CPI, whereas being burdened in only one area seems to make no big difference (*p* = 0.374; 95% CI [-9.2; 24.0]) (Fig. 2).

**Fig. 3:**
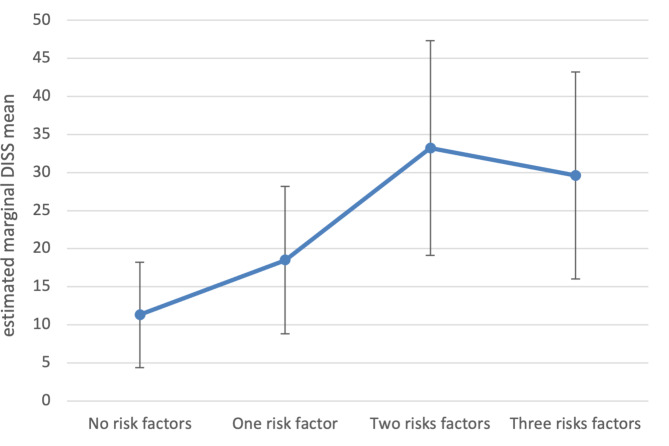
Estimated marginal means of pain-related disability (DISS) as a function of the number of stress types (excessive demands at work, work overload and social tensions). Controlled for age and sex. *N* = 68. Bars indicate 95% CI

Furthermore, ANCOVA reveals that the number of stress types (0, 1, 2 or 3; most associated stress types derived from hypothesis 1: excessive demands from work, work overload, social tensions) is also significantly related to DISS (F(3,60) = 3.5, *p* = 0.02, ω_p_²=0.109), whereas the covariates yield no significant difference. Planned contrasts reveal that, in comparison with no risk, having two (*p* = 0.008; 95% CI [5.8; 37.9]) or three (*p* = 0.021; 95% CI [2.9; 33.8]) of the most associated stress areas increases average DISS, whereas being burdened in only one area again seems to make no difference (*p* = 0.244; 95% CI [-5.0; 19.5]) (Fig. 3).

## Discussion

The current study detected that specific work-related stress types are associated with LBP strength and disability, and experiencing two or more of the stress types most related to LBP simultaneously leads to stronger and more disabling LBP among call-center workers.

Regarding hypothesis 1, the results suggest that, out of the 15 stress types under investigation, especially excessive demands at work and work overload seem to be associated with characteristic pain intensity and pain-related disability: Participants who experience a feeling of high demands at work display significantly stronger pain and impairment related to LBP; participants suffering from work overload experience significantly stronger impairment related to LBP. Thus, this study shows that call-center workers who experience specific work-related stressors (excessive demands at work and work overload) have a higher risk of developing LBP. Therewith, the current study extends the findings of three previous studies, which found a higher prevalence of neck and shoulder pain in VDU workers with job demands not matching their competence compared to VDU workers who had job demands which did match their competence [25], an association between high workload and general musculoskeletal disorders [22], and an association between work-related stress and LBP among a sample with intermittent back pain as part of the MiSpEx study [29]. Other studies have shown that the fear of negative social evaluation seems to trigger specific physiological alterations which could lead to increased chances of developing back pain problems [42, 43]. This assumption of the importance of social evaluation processes is supported by the comparison of the association of different stress types with LBP: It was assumed that, in comparison with non-work-related social and psychological stress types (e.g. social overload, social isolation, chronic worrying), especially work-related stress types influence the development of chronic LBP in call-center workers. This can partially be confirmed. In the current study, the work-related stress types “excessive demands from work” and “work overload” were found to be most closely associated with LBP. However, work discontent as a third work-related stress type doesn’t seem to be as strongly correlated; a surprising result, since a previous study found job satisfaction to be correlated with LBP [23] and the hypothesis assumed that all work-related variables should be influential. This difference to the work of Loghmani et al. (2013) may be caused by the fact that they investigated university employees, a population quite different from call-center employees. Moreover, the reason for the difference between the results of Loghmani et al. (2013) and the current study can also be due to the above-mentioned social evaluation processes: Whereas job demands and work overload are clearly associated with social evaluation (being able to fulfill job requirements), this is not the case for work discontent (which is more a feature of someone’s own evaluation of the job). To summarize, it is not sufficient to think of general stress when investigating the association between stress and specific disorders, it is also not sufficient to treat all stress subtypes in one stress domain as one factor. On the contrary, an even more detailed differentiation of work-related stress types seems to be necessary, as for example work-related stress types associated with social evaluation processes seem to be more influential than other work-related stress types.

When comparing work-related stress types with social (social overload, social tensions, and social isolation) and psychological (chronic worrying) stress types regarding their association with LBP, the strongest associations can be observed between work-related stress types and LBP. Additionally, we conducted Decision Trees anaylsis in SPSS which confirmed that excessive demands at work as well as work overload showed to be the most influential factors of the included stress factors in regards to LBP intensity and disability. Nevertheless, social tensions show the third strongest correlation with LBP, indicating that social support may also play a role in LBP intensity and disability among workers who sit for a prolonged time period, as already shown for the general population [44]. It is conceivable that strong social support can reduce the influence of negative social evaluation processes and weak support may increase it. According to these results, prevention and intervention programs should prioritize the reduction of work-related stress connected to social evaluation processes, and try to elevate social support on which employees can rely.

Regarding the second hypothesis, it could be shown that employees afflicted by two or three stress types, on average, suffer from stronger and more disabling LBP. These findings are supported by concepts discussed under the umbrella term of allostatic load [31, 45]: Increased stress exposure or an accumulation of stress leads to increased and prologend physiological activity of allosteric systems such as the immune system (e.g. inflammatory processes), slower recovery and, consequently, to more severe and disabling pain [8]. Remarkably, the pain burden is similar for people with no and with one stress risk factor. People with either two or three stress risk factors also have a similar pain burden. Especially the difference between having one or two risk factors seems to elevate perceived pain and impairment. Prevention and intervention programs aiming at stress reduction, therefore, do not necessarily need to diminish all sources of stress; a reduction of specific stress risk factors might yield similar good outcomes.

Concerning the results of both hypothesis 1 and 2, they can be influenced by additional factors such as the time participants have been working at the job. None of the included participants were in a trial period (6 months), which indicates that the TICS and ERI results, as well as the hair cortisol measurement, are based on the current job as call-center worker. Other work-related factors such as noise among the shared working space in a call-center as well as workplace ergnonomics can further contribute to the elevated stress levels among call-center workers.

A strength of the current study is that the data in this study were derived from two call-center locations, both with their own particular working procedure. Additionally, both research questions have not been investigated previously and are of high importance for the development of prevention and intervention programs for LBP in office surroundings. However, the results discussed above are also subject to a few limitations. Firstly, only 45% (*n* = 68) of the call-center workers working at the two locations could be measured. According to the power calculation made based on a pilot study, the sample size is acceptable, especially since only mean differences in CPI and DISS of at least 10 points (which equals an effect size of approximately *r* = 0.3) between the groups were considered as practically relevant [38]. Such differences can be discovered in the achieved sample size. Nevertheless, a repetition of the study with a bigger sample size might strengthen the results. Secondly, this was a study with a cross-sectional design and no longitudinal effects were measured. It may also be possible that low back pain causes stress or that both variables affect each other simultaneously. A longitudinal and/or quasi experimental research design would help to gain further insights into this question.

Lastly, although the most likely confounding variables (age, sex, education, income) were checked prior to the calculations, it cannot be ruled out completely that some of the observed differences are caused by unknown confounders or that existing differences are covered by these sociodemographic factors.

## Conclusion

Work-related stress, especially high demands at work and work overload, are associated with LBP, and should be specifically targeted in prevention programs aiming to reduce psychosocial risk factors in connection with LBP. Secondly, it was observed that especially employees aggregating two or more work-related stress types have elevated LBP intensity and disability. The results of the current study may be highly relevant for the development of LBP prevention programs for people within an office working context.

## Data Availability

The datasets used can be requested from the principal investigator with justification.

## Abbreviations

LBP: Low Back Pain
TICS: Trier Inventory for Chronic Stress
ERI: Effort Reward Imbalance Questionnaire
CPG: Chronic Pain Grade Questionnaire
CPI: Characteristic Pain Intensity
DISS: Pain-related Disability
BPI: Brief Pain Inventory
